# Spatial Orientation Impairment in Patients With Bilateral Vestibulopathy and Persistent Postural–Perceptual Dizziness

**DOI:** 10.1002/brb3.71025

**Published:** 2025-10-29

**Authors:** Vivien Oertle, Sandra Becker‐Bense, Thomas Brandt, Marianne Dieterich, Johannes Gerb

**Affiliations:** ^1^ German Center for Vertigo and Balance Disorders LMU University Hospital Munich Germany; ^2^ Graduate School of Systemic Neuroscience LMU Munich Munich Germany; ^3^ Department of Neurology LMU University Hospital Munich Germany; ^4^ Munich Cluster for Systems Neurology (SyNergy) Munich Germany

**Keywords:** 3D spatial pointing task, bilateral vestibulopathy, functional dizziness, persistent postural–perceptual dizziness, PPPD, spatial orientation

## Abstract

**Background:**

Two chronic forms of dizziness—bilateral vestibulopathy (BVP) with a loss of vestibular input, and functional dizziness with normal vestibular function—present with the key symptom of postural and gait imbalance. In BVP, this is associated with spatial disorientation. Here, we investigated whether persistent postural–perceptual dizziness (PPPD) in patients with normal vestibular function also affects spatial orientation, because there is evidence that central multisensory misintegration plays a crucial role in PPPD.

**Methods:**

Thirty‐two patients with BVP (mean age 52.44 ± 12.00 years; 17 females), 43 patients with PPPD (mean age 45.93 ± 11.72 years; 25 females), and 32 healthy controls (HC, mean age 44.78 ± 14.40 years; 15 females) participated in a clinical bedside test investigating spatial orientation abilities (three‐dimensional real‐world pointing task, 3D‐RWPT). This test includes a cognitive (mental rotation) and a vestibular paradigm (body rotation around yaw axis with eyes closed). Participants reported their perceived spatial abilities and levels of spatial anxiety /orientation‐related discomfort through standardized questionnaires.

**Results:**

Patients with BVP and PPPD showed significantly lower accuracy (i.e., larger angular deviations) in the 3D‐RWPT compared to HC (BVP: 9.62° ± 3.21°, PPPD: 9.16° ± 3.85°, HC: 7.77° ± 2.86°; *p* = 0.03), especially in the subtasks that rely on vestibular function (BVP: 8.11° ± 5.51°, PPPD: 6.62° ± 4.46°, HC: 4.45° ± 2.33°; *p* < 0.01). All cohorts had comparable levels of self‐assessed spatial abilities, while both BVP and PPPD patients showed higher levels of spatial orientation discomfort.

**Conclusions:**

This impairment of spatial orientation in PPPD patients with normal vestibular function could be a sign of (potentially anxiety‐driven) central suppression of vestibular input, which is required for the continuous updating of the internal representation of body motion and position relative to the environment.

## Graphical abstract

When asked to point at remembered targets before and after passive whole‐body rotations, patients with bilateral vestibular loss (BVP) and persistent postural–perceptual dizziness (PPPD) showed substantially decreased accuracy compared to healthy controls. In BVP patients, this is due to the complete vestibular loss; in PPPD patients, it could be a sign of disease‐dependent central suppression of vestibular input during head movements and locomotion.

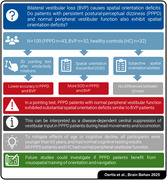

## 1 Introduction

The two most common chronic forms of dizziness in a specialized interdisciplinary tertiary center for vertigo and balance disorders are functional dizziness and bilateral vestibulopathy (BVP) (Strupp et al. 2023a). Functional dizziness is an umbrella term (Dieterich and Staab 2017), which covers different formerly separate entities such as phobic postural vertigo (Brandt 1996), chronic subjective dizziness (Staab et al. 2017), or visual vertigo (Bronstein 1995), as well as the current term of persistent postural–perceptual dizziness (PPPD) (Dieterich and Staab 2017; Staab et al. 2017). The key symptom of both conditions, PPPD and BVP, is a distressing instability of stance and gait. In BVP patients, it is furthermore well recognized that the loss of vestibular function affects spatial orientation, as shown in rodents (Smith et al. 2005; Smith et al. 2015) as well as in humans (Brandt et al. 2005; Kremmyda et al. 2016; E. S. Lee et al. 2023; Dobbels et al. 2019). This was supported in other studies by significantly decreased spatial performance and higher spatial anxiety scores in BVP patients (Kremmyda et al. 2016; Elyoseph et al. 2023). While PPPD patients with an intact vestibular system also complain about subjective dizziness, the effect of PPPD on patients’ spatial orientation and navigation skills has not yet been sufficiently examined. Virtual reality (VR) studies by Breinbauer et al. (2023, 2020) described an impairment of spatial orientation in PPPD; however, their results cannot be unequivocally assigned to PPPD alone because the patients investigated in their studies partially suffered from various additional vestibular disorders, including lasting peripheral vestibular dysfunction.

Numerous studies could demonstrate how postural instability in BVP patients increases if vision or somatosensory input cannot compensate for the vestibular loss, for example, when walking in darkness or on uneven ground (Schniepp et al. 2017). An impairment of balance control has also been demonstrated neurophysiologically in PPPD, characterized by an increased high‐frequency body sway at normal stance independent of visual input (Querner et al. 2000; Krafczyk et al. 1999; Holmberg et al. 2003). Postural sway in PPPD patients, in contrast to BVP patients, however, improves in more challenging balance conditions, for example, during tandem stance with closed eyes (Querner et al. 2000). The postural instability in PPPD is likely caused by an application of an inadequate balance strategy with nonphysiological muscular co‐contraction, a postural behavior that healthy persons use when performing difficult balance tasks (Carpenter et al. 2001). Stabilogram diffusion analysis showed an inadequate interaction between open‐ and closed‐loop postural control, potentially due to a lowered sensory feedback threshold elicited by an anxiously conscious balance control (Wuehr et al. 2013). Since this affects sensorimotor integration of different modalities (vestibular, visual, and somatosensory), we were interested in whether PPPD patients show an impairment of spatial orientation similar to that demonstrated in BVP.

The three‐dimensional real‐world pointing task (3D‐RWPT; Gerb et al. 2023a) was used because it provides a simple measure of spatial memory and abilities, based on a simple sensorimotor activity (whole‐arm pointing). In short, the 3D‐RWPT requires the seated participant to update their mental representation of the environment following horizontal whole‐body rotations around the yaw axis to correctly interact with remembered static real‐world targets. In previous studies, the reliability of 3D‐RWPT with respect to the impact of participants’ sex and age (Gerb et al. 2023a), groupwise effects of cognition and bilateral vestibular dysfunction (Gerb et al. 2024a), and test performance correlation with spatial orientation discomfort/spatial anxiety scores (Gerb, Oertle, et al. 2024) was demonstrated.

In the current study, we examined PPPD patients with neurotologically proven normal peripheral vestibular function using the 3D‐RWPT and compared their performance to (i) patients with BVP without functional dizziness and (ii) age‐matched healthy controls. To mitigate potential age effects, only patients younger than 65 years were recruited. To avoid potential biases by cognitive impairment, which can also affect spatial orientation and memory (Gerb et al. 2024a), only patients with normal scores in a dementia screening test were included. The major question was whether the differential effects of reduced vestibular input in BVP and the potentially disease‐related central suppression of vestibular input in PPPD patients with an intact peripheral vestibular system have similar or different effects on spatial orientation and particularly on specific subtests.

## 2 Methods

### 2.1 Patients

All patients and healthy controls were examined in the interdisciplinary German Center for Vertigo and Balance Disorders (DSGZ), LMU University Hospital, Munich, Germany, between September 2020 and September 2024. Diagnoses were made according to the international diagnostic criteria of the Bárány‐Society (Staab et al. 2017; Strupp et al. 2017). Inclusion criteria were an age between 18 and 65 years, normal scores in a dementia screening test (Montreal Cognitive Assessment [MoCA; Nasreddine et al. 2005], after correction for education level), normal hearing function, and (for the patients) a diagnosis made by an experienced neurotologist. A priori sample size calculation was conducted using G*Power (v3.1; Faul et al. 2007), based on a large effect size (*f* = 0.40), power = 0.95, and *α* = 0.05, indicating a required minimum sample size of 102.

The study was performed in accordance with the ethical standards laid down in the 1964 Declaration of Helsinki and its later amendments. The data protection clearance and the Institutional Review Board of the Ludwig‐Maximilians‐University, Munich, Germany, approved the study (no. 094–10), and all patients gave informed consent.

### 2.2 Clinical and Neurotological Testing

Clinical testing included a neurological and neuro‐orthoptic examination (Strupp et al. 2023b), that is, spontaneous and head‐shaking nystagmus, ocular motor examination, fundus photography and adjustment of the subjective visual vertical (SVV, in order to detect central vestibular deficits and acute vestibular tonus imbalances; Brandt and Dieterich 2017; Dieterich and Brandt 1993), video‐oculography during bithermal water caloric testing (to measure the function of the horizontal semicircular canals in the low‐frequency range of the vestibulo‐ocular reflex), and standardized video‐head‐impulse‐test measurements of the semicircular function in the high‐frequency range using the EyeSeeCam system (EyeSeeTec, Munich, Germany). In addition, postural function of PPPD patients was determined using clinical routine posturography on a stabilometer platform (Type 9261A; Kistler, Winterthur, Switzerland) in conjunction with a dedicated neural network analysis (see details in Krafczyk et al. 2006). Briefly, in this assessment, the sway pattern of the patients is measured in 10 increasingly challenging stance conditions (including standing with open and closed eyes, on foam, with head reclination, and in tandem stance). Subsequently, using an artificial neural network, an automated diagnostic assignment to healthy stance behavior or common postural disorders (including functional stance in PPPD) is made.

### 2.3 Psychometric Testing

The psychometric questionnaire battery consisted of (i) the Santa Barbara Sense of Direction Scale (SBSODS; Hegarty et al. 2002), (ii) the Patient Health Questionnaire subsection 9 (PHQ‐9; Kroenke et al. 2001), (iii) the Edinburgh Handedness Inventory (EHI; Oldfield 1971), (iv) MoCA (Nasreddine et al. 2005), (v) the Extended Inventory for Spatial Orientation Discomfort (EISOD; Gerb, Oertle, et al. 2024), and (vi) the German version of the state/trait anxiety inventory (STAI) in its short version (Spielberger et al. 1971). SBSODS, PHQ‐9, EHI, and STAI were filled out by the participants themselves without supervision or time constraints, while the MoCA screening test (Nasreddine et al. 2005) was performed in a standardized fashion by a medical doctor or a trained doctoral student.

### 2.4 Three‐Dimensional RWPT

**FIGURE 1:**
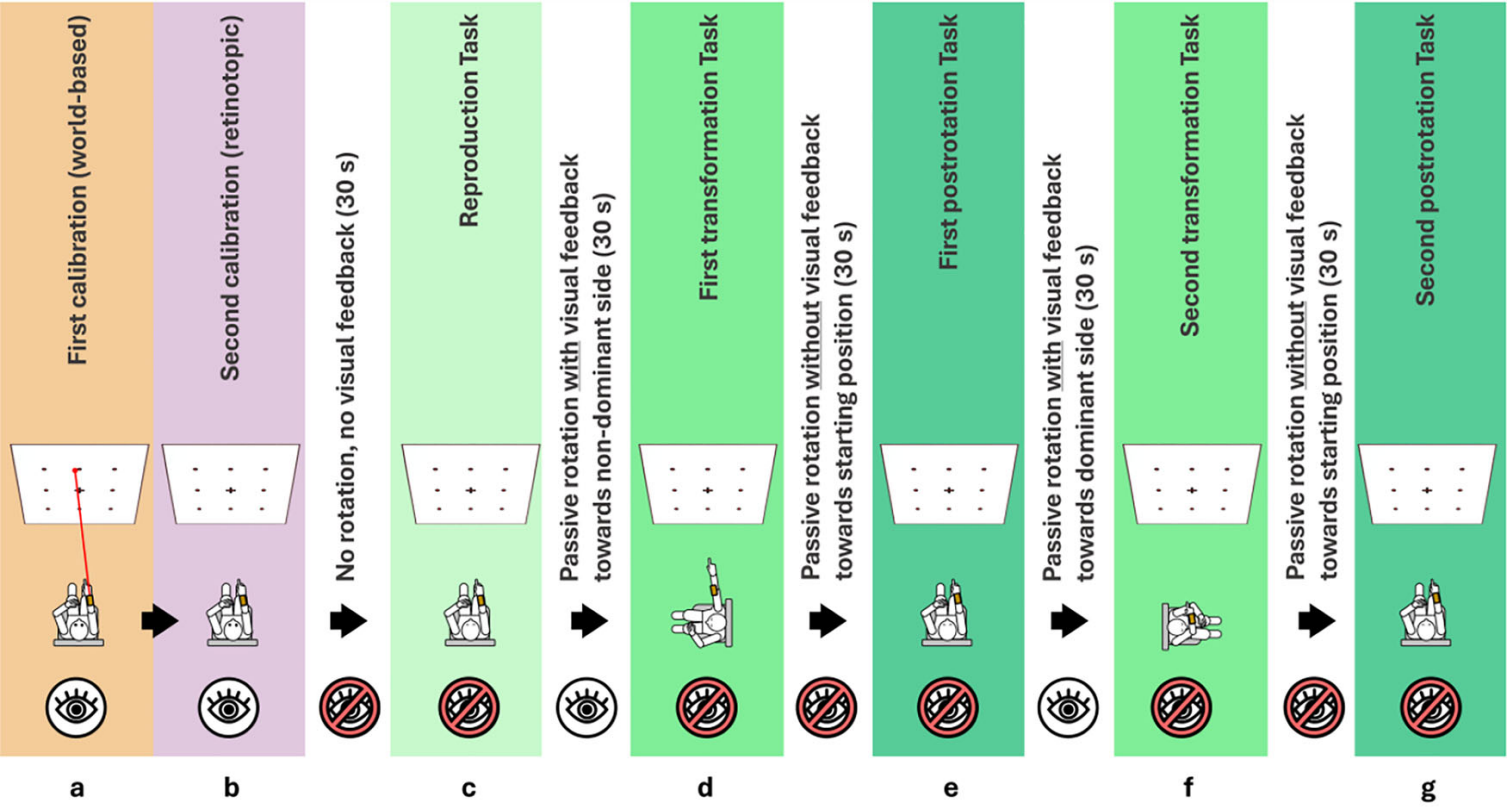
Overview of the 3D‐RWPT. The full test consists of two calibration paradigms (a, b) and five test paradigms (c–g). Note that the first whole‐body rotation was always performed in the direction of the non‐hand‐dominant side, that is, toward the left side for right‐handed participants and toward the right for left‐handed participants.

The clinical pointing task was recorded using a smartphone‐based pointing device (Flanagin et al. 2019) and the testing setup from multiple previous studies (Gerb et al. 2023a, 2023b, 2024a, 2024b; Gerb, Oertle, et al. 2024). The 3D‐RWPT includes two calibration and five testing paradigms, which the participant performs while seated on a swivel chair with their eye level aligned with the center row of a 3 × 3 rectangular target matrix (marked using well‐visible dots on a wall at a distance of 192 cm). For each task, a computerized voice from the pointing device attached to the participants forearm gives a command, for example, “top left,” and the subjects points toward the target with their extended dominant (as determined by the EHI) arm. The calibration is performed once for world‐based spatial encoding (with a laser pointer attached to the device, visualizing the real‐world pointing vector; Figure 1a) and once for retinotopic spatial encoding (i.e., without the laser pointer, so participants visually align the target and extended index finger; Figure 1b). Participants with overly inaccurate calibrations are excluded from further analysis. Afterward, subjects are asked to point to the targets in a newly randomized order without visual feedback while facing straight ahead (Figure 1c), after being passively 90° rotated to their non‐hand‐dominant side (Figure 1d), back in the initial position (Figure 1e), after being passively rotated 90° to their hand‐dominant side (Figure 1f), and back in the initial target‐facing position (Figure 1g). Note that during all pointing paradigms, participants have their eyes closed and rest their back against the chair.

### 2.5 Three‐Dimensional RWPT Analysis

The pointing vectors from each 3D‐RWPT paradigm were used to calculate mean angular deviations (lowest possible value: 0°) in the azimuth (horizontal) and polar (vertical) planes relative to the two sets of calibrations, as described in previous studies (Gerb et al. 2023a). In short, accelerometer data from the pointing device were used to determine the pointing angle in spherical coordinates. Since the center of the spherical coordinate system does not change between paradigms (due to the unchanged axis of rotation), plane‐specific angular deviations can be calculated directly (for a more detailed description, see Gerb et al. 2023a, 2024a). The preferred spatial encoding strategy was calculated as described (Gerb et al. 2024a). For this, first paradigm‐specific angular deviations from either retinotopic or world‐based calibration (Gerb et al. 2022) were calculated. Afterward, the deviation using the world‐based calibration was subtracted from the deviation using the retinotopic calibration, resulting in negative values for predominantly retinotopic/egocentric spatial encoding and positive values for predominantly world‐based/allocentric spatial encoding. In order to approximate the “level of disorganization” of each pointing performance, we analyzed the average standard deviation of pointing vectors in all paradigms: more disorganized pointing performances, that is, a larger pointing spread, resulted in a higher value in this metric.

### 2.6 Statistical Analysis

After data collection, all data were anonymized and processed using Microsoft Excel (Version 2025) and JASP (Version 0.18.3, jasp‐stats.org). For descriptive data analyses, mean values and standard deviation were used for continuous variables and absolute frequencies were used for categorical variables. We tested statistical inference using Spearman's rho and performed either independent samples student's *t*‐test or one‐way analysis of variance (ANCOVA) testing when comparing the cohorts (correcting for respective covariates depending on subanalyses, with post hoc testing using 1000 bootstraps and Tukey correction; planned contrasts: patient cohorts; all analyses performed in JASP). For nonparametric variables (determined by Levene's testing), Kruskal–Wallis tests and Dunn's post hoc tests were used.

## 3 Results

Forty‐three patients with PPPD (25 females), 32 patients with BVP (17 females), and 32 healthy controls (15 females) were enrolled. The groups diverged in their average age (ANOVA mean age: *F*(102, 2) = 3.51, *p* = 0.03; mean age BVP: 52.44 ± 12.00 years, PPPD: 45.93 ± 11.72 years, HC: 44.78 ± 14.40 years). Post hoc testing revealed significant differences between BVP and HC (*p* = 0.05), but not between the other cohorts. In order to account for these age differences, all other analyses were corrected for patient age.

No significant group differences existed for patient MoCA scores (ANCOVA: n.s.; mean MoCA BVP: 28.31 ± 1.34 points, PPPD: 28.35 ± 1.55 points, HC: 28.40 ± 1.43 points).

To ensure normal vestibular function in the HC cohort, all participants underwent detailed neurotological testing. As expected, the groups did diverge in their mean caloric excitability (ANOVA mean caloric slow phase velocity [SPV]: *F*(88, 2) = 46.91, *p* < 0.001; mean SPV BVP: 1.74 ± 1.31°/s, PPPD: 20.00 ± 10.84°/s, HC: 19.59 ± 8.01°/s). Post hoc testing confirmed that only the BVP cohort had significantly lower caloric excitability (BVP vs. HC: *p* < 0.001; BVP vs. PPPD: *p* < 0.001), while PPPD and HC had comparable peripheral vestibular function. Similarly, the groups exhibited different vHIT gains (ANOVA right vHIT: *F*(90, 2) = 140.24, *p* < 0.001; mean gain BVP: 0.25 ± 0.20, PPPD: 0.97 ± 0.21, HC: 0.89 ± 0.10; ANOVA left vHIT: *F*(90, 2) = 137.30, *p* < 0.001; mean gain BVP: 0.29 ± 0.25, PPPD: 0.98 ± 0.16, HC: 0.94 ± 0.12). Again, post hoc testing showed that the BVP cohort had significantly lower gain values on both sides (BVP vs. HC: *p* < 0.001; BVP vs. PPPD: *p* < 0.001), while PPPD and HC both had normal peripheral vestibular function.

Patient demographics can be seen in table 1.

**TABLE 1:** Patient demographics and neurotological testing results. Avoidance behavior and symptom aggravation by visual stimuli were assessed by an experienced neurotologist by structured history‐taking.

	BVP	PPPD	HC	Statistical analysis (ANOVA)	Notes
***N* (of which female, %)**	32 (17, 53.1%)	43 (25, 58.1%)	32 (15, 46.9%)	—	
**Age (years)**	52.44 ± 12.00	45.93 ± 11.72	44.78 ± 14.40	*p* = 0.03	No significant difference between PPPD and HC cohort (Kruskal–Wallis test)
**MoCA (points)**	28.31 ± 1.34	28.35 ± 1.55	28.40 ± 1.43	n.s.	
**Caloric excitability (in °/s)**	1.74 ± 1.31	20.00 ± 10.84	19.59 ± 8.01	*p* < 0.001	No significant difference between PPPD and HC cohort (Kruskal–Wallis test)
**vHIT gain (right side, at 60 ms)**	0.25 ± 0.20	0.97 ± 0.21	0.89 ± 0.10	*p* < 0.001	No significant difference between PPPD and HC cohort (Kruskal–Wallis test)
**vHIT gain (left side, at 60 ms)**	0.29 ± 0.25	0.98 ± 0.16	0.94 ± 0.12	*p* < 0.001	No significant difference between PPPD and HC cohort (Kruskal–Wallis test)
**PPPD disease duration in months (min, max)**	—	29.14 ± 41.02 (1, 170)	—	—	PPPD diagnostic criteria require symptoms for at least three months. Disease duration refers to time (in months) since the earliest possible time of PPPD diagnosis, i.e., three months after symptom onset.
**Relevant avoidance behavior (%)**	—	27 (62.8%)	—	—	—
**Symptom aggravation through visual stimuli (%)**	—	25 (58.1%)	—	—	—

### 3.1 Three‐Dimensional RWPT Results

#### 3.1.1 Accuracy Analysis

As a first step, significant group differences in the calibration accuracy (which would render further analyses impossible) were ruled out (ANCOVA: n.s.).

In the polar direction, no group differences in any of the 3D‐RWPT paradigms were observable (ANCOVA overall accuracy, reproduction accuracy, transformation accuracy, and post‐rotation accuracy: all n.s.; post hoc testing: all n.s.). The following analyses therefore refer to the azimuth (horizontal) plane, that is, the direction of body rotation.

**FIGURE 2:**
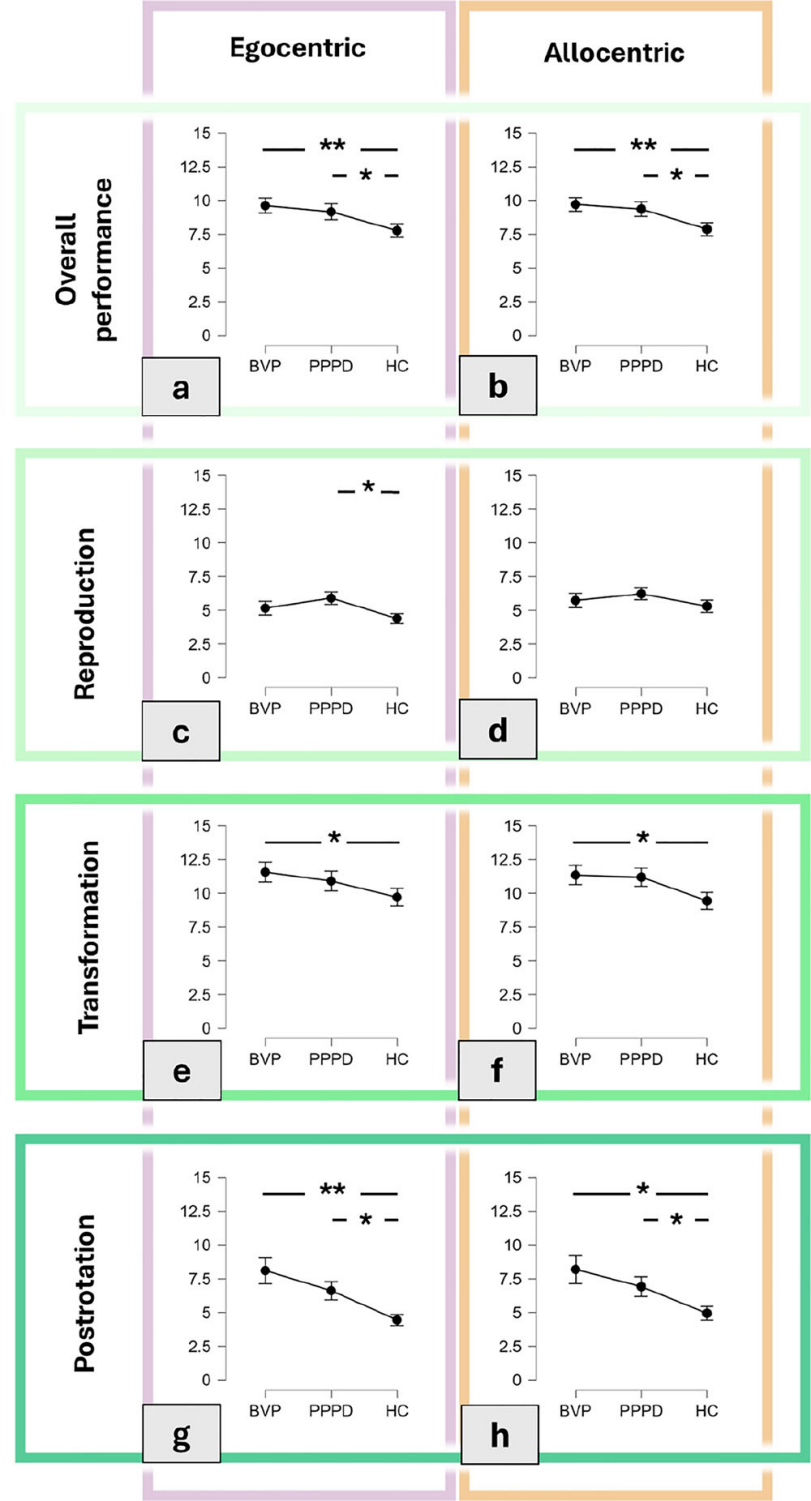
Angular accuracy in the 3D‐RWPT, calculated from retinotopic/egocentric calibration (purple box) and allocentric/world‐based calibration (orange box). Lower angular deviations represent higher accuracy. All graphs depict the azimuth (horizontal) deviation. From top to bottom: (a, b) overall performance (all testing paradigms); (c, d) initial reproduction paradigm; (e, f) transformation paradigm; and (g, h) post‐rotation paradigm. The BVP cohort showed the largest deviations compared to HC, especially noticeable in the post‐rotation paradigm (g, h), but also in the transformation paradigm (e, f). In the PPPD cohort, significant deviations were observable partially in the reproduction paradigm (c) and more robustly in the post‐rotation paradigm (g, h). BVP, bilateral vestibulopathy; PPPD, persistent postural–perceptual dizziness; HC, healthy controls. Significant comparisons are marked with asterisks (*p < 0.05, **p < 0.01); nonsignificant post hoc comparisons are not marked.

In their overall accuracy (i.e., averaged accuracy from all five paradigms), the HC cohort outperformed both other cohorts in the egocentric/retinotopic (age‐corrected ANCOVA for mean accuracy: *F*(104, 2) = 2.63, *p* = 0.08; mean deviation BVP: 9.62° ± 3.21°, PPPD: 9.16° ± 3.85°, HC: 7.77° ± 2.86°; planned contrasts post hoc analysis: BVP vs. HC: *p* < 0.01, BVP vs. PPPD: *p* = 0.38, PPPD vs. HC: *p* = 0.04; Figure 2a) and the allocentric/world‐based analysis (age‐corrected ANCOVA for mean accuracy: *F*(104, 2) = 3.13, *p* = 0.05; mean deviation BVP: 9.71° ± 2.84°, PPPD: 9.36° ± 3.60°, HC: 7.88° ± 2.75°; planned contrasts post hoc analysis: BVP vs. HC: *p* < 0.01, BVP vs. PPPD: *p* = 0.42, PPPD vs. HC: *p* = 0.05; Figure 2b).

Additionally, we analyzed all individual paradigms. In the initial reproduction, the PPPD cohort showed higher deviations than both other groups, only reaching statistical significance in the egocentric analysis (age‐corrected ANCOVA for mean egocentric accuracy: *F*(104, 2) = 2.85, *p* = 0.06; mean deviation BVP: 5.16° ± 2.88°, PPPD: 5.90° ± 3.02°, HC: 4.39° ± 2.06°; planned contrasts post hoc analysis: BVP vs. HC: *p* = 0.34, BVP vs. PPPD: *p* = 0.32, PPPD vs. HC: *p* = 0.04; ANCOVA allocentric accuracy: *F*(104, 2) = 0.99, *p* = 0.37; mean deviation BVP: 5.73° ± 3.01°, PPPD: 6.23° ± 2.88°, HC: 5.30° ± 2.54°; planned contrasts post hoc analysis: BVP vs. HC: *p* = 0.82, BVP vs. PPPD: *p* = 0.28, PPPD vs. HC: *p* = 0.18; Figure 2c,d).

In the (cognitively demanding) transformation paradigm, the HC cohort outperformed both other cohorts in the egocentric/retinotopic (age‐corrected ANCOVA for mean accuracy: *F*(104, 2) = 1.56, *p* = 0.21; mean deviation BVP: 11.57° ± 5.16°, PPPD: 10.91° ± 4.78°, HC: 9.71° ± 3.65°; planned contrasts post hoc analysis: BVP vs. HC: *p* = 0.05, BVP vs. PPPD: *p* = 0.34, PPPD vs. HC: *p* = 0.24; Figure 2e) and the allocentric/world‐based analysis (age‐corrected ANCOVA for mean accuracy: *F*(104, 2) = 2.27, *p* = 0.11; mean deviation BVP: 11.35° ± 4.01°, PPPD: 11.20° ± 4.46°, HC: 9.44° ± 3.49°; planned contrasts post hoc analysis: BVP vs. HC: *p* = 0.04, BVP vs. PPPD: *p* = 0.73, PPPD vs. HC: *p* = 0.06; Figure 2f).

In the postrotation paradigm (which requires vestibular sensory processing), the HC cohort again outperformed both other cohorts in the egocentric/retinotopic (age‐corrected ANCOVA for mean accuracy: *F*(104, 2) = 5.80, *p* < 0.01; mean deviation BVP: 8.11° ± 5.51°, PPPD: 6.62° ± 4.46°, HC: 4.45° ± 2.33°; planned contrasts post hoc analysis: BVP vs. HC: *p* < 0.01, BVP vs. PPPD: *p* = 0.33, PPPD vs. HC: *p* = 0.01; Figure 2g) and the allocentric/world‐based analysis (age‐corrected ANCOVA for mean accuracy: *F*(104, 2) = 3.89, *p* = 0.02; mean deviation BVP: 8.19° ± 5.91°, PPPD: 6.92° ± 4.78°, HC: 4.94° ± 2.90°; planned contrasts post hoc analysis: BVP vs. HC: *p* = 0.01, BVP vs. PPPD: *p* = 0.59, PPPD vs. HC: *p* = 0.03; Figure 2h).

#### 3.1.2 Qualitative Aspects: Pointing Strategy, Pointing Disorganization

No group differences in the employed pointing strategy were found in azimuth direction or polar direction. The spread of pointing vectors (as an approximation of participant pointing disorganization) during the experiment was typically the highest in the PPPD cohort, although without reaching statistical significance (ANCOVA: n.s.; mean retinotopic azimuth spread BVP: 6.29°, PPPD: 7.12°, HC: 6.08°; mean world‐based azimuth spread BVP: 6.09°, PPPD: 6.64°, HC: 5.89°; mean retinotopic polar spread BVP: 5.43°, PPPD: 6.02°, HC: 6.09°; mean world‐based polar spread BVP: 4.55°, PPPD: 5.42°, HC: 4.97°).

### 3.2 Psychometric Results

#### 3.2.1 PHQ‐9

The groups differed substantially in the depression screening test (ANCOVA PHQ‐9 score: *F*(100, 2) = 8.99, *p* < 0.001; mean score BVP: 4.61 ± 3.28, PPPD: 7.79 ± 5.82, HC: 3.40 ± 3.60). Post hoc testing showed similar scores in the BVP and HC cohorts (BVP vs. HC: n.s.), while PPPD patients had higher scores than both other subgroups (BVP vs. PPPD: *p* = 0.01; PPPD vs. HC: *p* < 0.001).

#### 3.2.2 SBSODS

No group differences were observable in the self‐assessed spatial abilities in the SBSODS (ANCOVA: n.s.; overall scores: BVP: 4.69 ± 0.98 points, PPPD: 4.76 ± 0.99 points, HC: 4.58 ± 1.12 points).

#### 3.2.3 STAI

The groups differed substantially in their trait anxiety levels (ANCOVA: *F*(53, 2) = 3.91, *p* = 0.03; mean score BVP: 27.47 ± 12.37, PPPD: 40.25 ± 17.68, HC: 31.22 ± 9.46). Post hoc testing revealed a significant difference between PPPD and BVP (*p* = 0.03). Similarly, the groups differed in their state anxiety levels (ANCOVA: *F*(53, 2) = 4.05, *p* = 0.02; mean score BVP: 34.62 ± 17.31, PPPD: 42.81 ± 17.79, HC: 27.04 ± 16.39). Post hoc testing revealed a significant difference between PPPD and BVP (*p* = 0.02).

#### 3.2.4 EISOD

**FIGURE 3:**
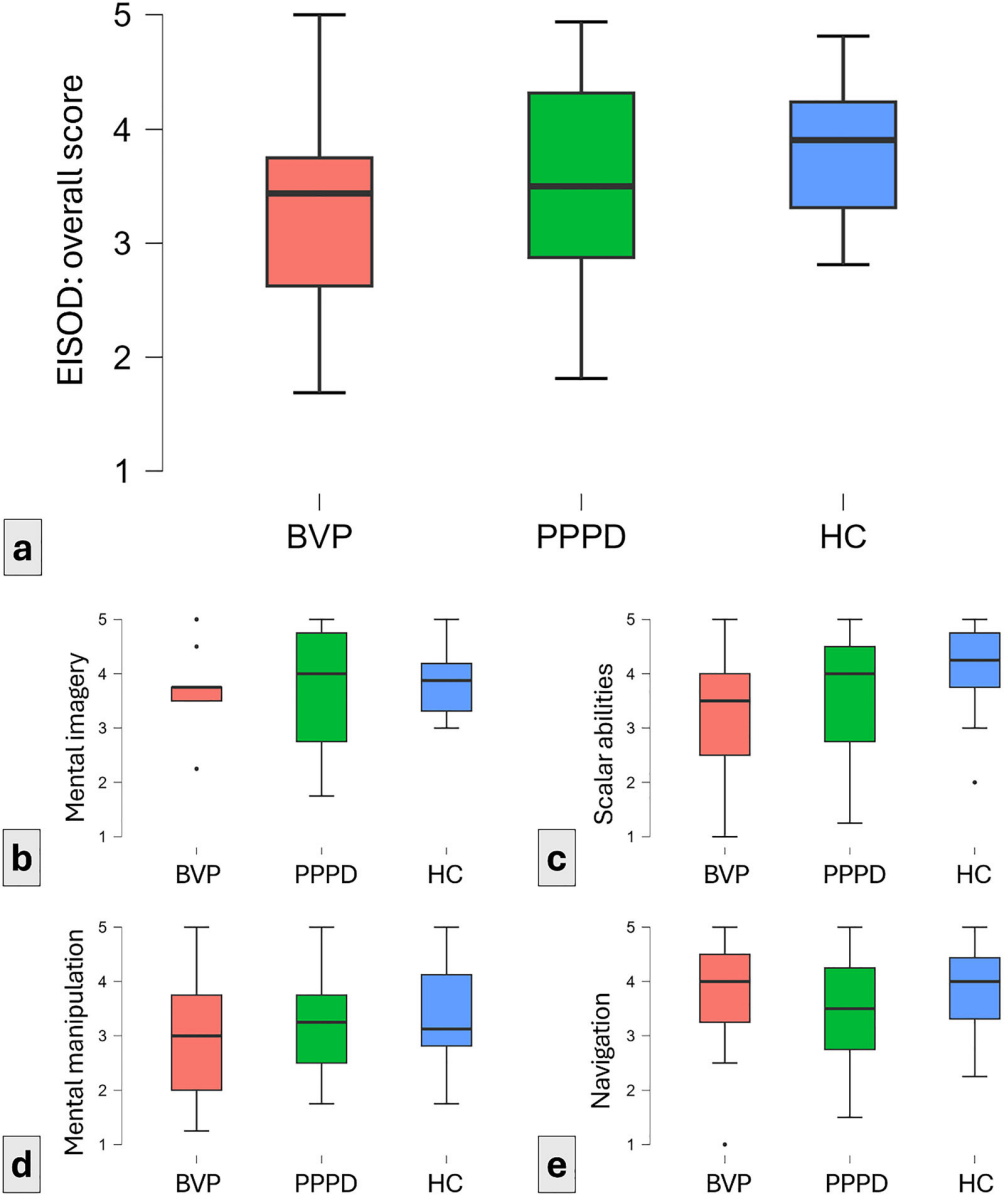
Boxplots of average EISOD scores. Here, the HC cohort (blue box) had the highest average score, indicating a lower rate of spatial orientation discomfort than PPPD (blue box) and BVP (red box). From left to right: (a) overall EISOD scores; (b) mental imagery subscore; (c) scalar abilities subscore; (d) mental manipulation subscore; and (e) navigation subscore. BVP, bilateral vestibulopathy; PPPD, persistent postural–perceptual dizziness; HC, healthy controls.

Without reaching statistical significance (ANCOVA, corrected for state and trait anxiety levels: n.s.), the HC cohort had the lowest rate of spatial orientation discomfort (i.e., the highest scores in the EISOD; BVP: 3.39 ± 0.95 points, PPPD: 3.53 ± 0.84 points, HC: 3.81 ± 0.62 points; Figure 3a). This pattern was observable in the mental imagery subscore (BVP: 3.69 ± 0.84 points, PPPD: 3.69 ± 1.06 points, HC: 3.88 ± 0.65 points; Figure 3b), scalar abilities (BVP: 3.19 ± 1.17 points, PPPD: 3.59 ± 1.20 points, HC: 4.07 ± 0.87 points; Figure 3c), and mental manipulation (BVP: 2.96 ± 1.24 points, PPPD: 3.34 ± 0.86 points, HC: 3.45 ± 0.94 points; Figure 3d). In the navigation subset, PPPD patients reported the lowest scores, that is, more pronounced spatial orientation discomfort (BVP: 3.71 ± 1.14 points, PPPD: 3.51 ± 1.07 points, HC: 3.84 ± 0.84 points; Figure 3e).

### 3.3 Effect of Clinical Symptoms

We found no significant correlation between disease duration and 3D‐RWPT accuracy, SBSODS scores, or EISOD scores (Spearman's rho: n.s.). Group comparisons yielded no significant differences for 3D‐RWPT accuracy, SBSODS scores, or EISOD scores when assessing patients’ avoidance behavior (Welch's *t*‐test: n.s.), symptom aggravation through visual stimuli (Welch's *t*‐test: n.s.), or a PPPD‐typical swaying pattern in posturography (functional swaying pattern in 14 patients, Welch's *t*‐test: n.s.; note that 10 PPPD patients did not undergo posturography).

### 3.4 Effect of Anxiety Levels on Post‐Rotation Angular Accuracy

Independent of diagnosis, world‐based (but not retinotopic) post‐rotation angular deviation correlated with state (Spearman's rho: 0.37, *p* = 4.98 × 10^−3^, Fisher's *z* = 0.39) and trait anxiety levels (Spearman's rho: 0.28, *p* = 0.03, Fisher's *z* = 0.29). Higher spatial orientation discomfort regarding scalar abilities (i.e., lower EISOD scores in the scalar abilities subset) was associated with worse performance (Spearman's rho, retinotopic calibration: −0.36, *p* = 6.74 × 10^−3^, Fisher's *z* = −0.37; world‐based calibration: −0.27, *p* = 0.04, Fisher's *z* = 0.28).

### 3.5 Effect of Depression Levels on Pointing Accuracy

Scores in the depression screening test (PHQ‐9) showed no significant correlation with pointing accuracy in any of the 3D‐RWPT paradigms (Spearman's rho: all n.s.).

### 3.6 Effect of Anxiety Levels on Spatial Encoding Strategy

Higher state (Spearman's rho: −0.39, *p* = 2.99 × 10^−3^, Fisher's *z* = −0.41) and trait anxiety levels (Spearman's rho: −0.30, *p* = 0.03, Fisher's *z* = −0.30) were associated with more retinotopic/egocentric spatial encoding in the horizontal but not the vertical plane.

## 4 Discussion

Patients with PPPD exhibited a similar, albeit less pronounced, pattern of visuospatial impairment as patients with BVP in a bedside sensorimotor pointing test for spatial abilities (3D‐RWPT), with both groups showing higher deviations (i.e., lower accuracy) than age‐matched healthy controls. Substantial deficits were seen particularly in the post‐rotation 3D‐RWPT paradigms with closed eyes, which require the correct integration of vestibular and somatosensory input about the angle of horizontal body rotation relative to the environment. Both BVP and PPPD patients reported higher subjective spatial orientation discomfort (EISOD) than the control participants, while all three groups had comparable levels of self‐assessed spatial abilities (SBSDOS). The latter finding could be explained by the fact that the patients assessed their lifelong orientation abilities compared to others, rather than their currently limited disease‐related performance; self‐assessed spatial abilities alone have been shown to not reliably correlate with vestibular function (Gerb et al. 2024b).

The visuospatial deficits in the BVP control cohort are in line with previous research (Smith et al. 2015; Dobbels et al. 2019; Gerb et al. 2024a; Zwergal et al. 2024). Their post‐rotation deficits can be explained by the lack of labyrinthine input during the rotation without visual feedback (Anson et al. 2021). As a compensatory strategy in bilateral vestibular loss, sensory reweighting is necessary, with greater reliance on, for example, the visual system (Medendorp et al. 2018). It is therefore remarkable that PPPD patients struggled the most in the same paradigm (i.e., following a passive whole‐body yaw‐axis rotation without visual feedback) despite their intact peripheral vestibular function. Thus, a central sensory suppression or misintegration of vestibular input in PPPD patients can be hypothesized. In the current study, further evidence for this hypothesis can be derived from the fact that all observed group differences were found in the azimuth (horizontal) plane, that is, the plane of the (vestibular) stimulus, while no group differences were observed in the polar (vertical) plane. Note that this is in line with a previous study by our group, which found that human horizontal spatial orientation is directly affected by aging, cognitive impairment, or vestibular loss, while vertical spatial orientation seems to be unaffected (Gerb et al. 2025).

In functional dizziness, for example, phobic postural dizziness, anxiety‐related personality traits (Eckhardt‐Henn et al. 2008; Staab et al. 2014), psychiatric comorbidity with anxiety disorders and depression (Lahmann et al. 2015; Best et al. 2009), and altered postural control strategies (Carpenter et al. 2001; Wuehr et al. 2013; Münchener and Dieterich 1986; Guerraz et al. 2001) were described, further supporting the hypothesis of a (potentially anxiety‐driven) misintegration of vestibular sensory information as a key feature of PPPD. Accordingly, fMRI studies demonstrated increased activation of emotional networks (Popp et al. 2018) and abnormal sensory integration in PPPD cohorts (Popp et al. 2018; Na et al. 2019; J. O. Lee et al. 2018; Im et al. 2021; Li et al. 2025; Huber et al. 2020), interpreted as inadequate interactions between the cingulo‐opercular network, attention network, auditory network, and visual network. These inadequate interactions might also play a role in the development of spatial impairment in PPPD patients, although more detailed fMRI studies are needed. Potentially, future neuroimaging studies in PPPD patients could explicitly analyze changes in spatial processing networks, for example, by having participants perform spatial experiments during fMRI.

Other psychophysical studies found disturbed vestibulo‐perceptive thresholds in PPPD patients (Wurthmann et al. 2021; Storm et al. 2024; Helmchen et al. 2024). For example, in one study, the perception threshold of galvanic vestibular stimulation was lower in PPPD patients than in healthy controls, but the threshold of correctly perceived egomotion during chair rotation did not differ (Wurthmann et al. 2021). The findings of a lower threshold for the perception of visually induced apparent self‐motion of rollvection and less compensatory lateral postural sway in phobic postural vertigo (Querner et al. 2002) and the lower thresholds of the perception of galvanic vestibular stimulation (Wurthmann et al. 2021; Storm et al. 2024) can be explained most convincingly by a central “compensatory” mechanism aimed to suppress changes in body position in space caused by visual and vestibular motion stimuli. This is in agreement with prior studies in patients with phobic postural vertigo, where external perturbations of body position were linked to an increased subjective disturbance of balance (Querner et al. 2000, 2002).

As an additional factor relevant for PPPD pathogenesis, prior studies investigated visual dependency, which is increased in PPPD patients (Bronstein 1995; Guerraz et al. 2001; De Vestel et al. 2022). It therefore has been postulated before that PPPD patients might rely more on visual cues than on vestibular cues during spatial encoding of their environment, potentially due to altered visual cortical processing (Passamonti et al. 2018; Riccelli et al. 2017). Potentially, increased visual dependency might constitute a direct compensatory reaction to disturbed vestibular sensory perception.

In our experiments, both PPPD and BVP patients exhibited higher rates of spatial orientation discomfort, while PPPD patients reported the highest state and trait anxiety levels. This is in line with prior investigations in BVP patients (E. S. Lee et al. 2023) and PPPD patients (Jáuregui‐Renaud et al. 2024). In a previous study conducted in healthy participants, anxiety levels were linked to the ability to update orientation in the horizontal plane following whole body rotations (Alcantara‐Thome et al. 2021). In our study (which involved a comparable experimental setup), similar findings were observed: higher state and trait anxiety levels as well as more pronounced spatial orientation discomfort were associated with worse post‐rotational angular accuracy.

As a limitation of the current study, the slightly unequal age distribution has to be addressed. Despite only including participants under the age of 65 years in the study, the BVP cohort had a higher average age then the PPPD cohort and the healthy controls. To mitigate potential age effects, all statistical analyses were age‐corrected. Furthermore, it should be noted that the PPPD disease duration range was substantial, ranging from only 4 months to more than 14 years since symptom onset. While no correlation between disease duration and spatial impairment severity was found, further research is needed to understand the time course of spatial disorientation in PPPD patients, for example, if it constitutes an immediate symptom of the disorder (potentially even preceding dizziness), or if it develops over time. Lastly, future studies investigating spatial impairment in PPPD could aim to combine behavioral measures (such as the 3D‐RWPT) with VR experiments (Breinbauer et al. 2023, 2020) and neuroimaging in order to fully understand how PPPD affects spatial cognition.

## 5 Conclusion

PPPD patients with normal peripheral vestibular function and BVP patients with chronic peripheral vestibular loss both exhibited significant spatial orientation deficits in a bedside pointing task for spatial orientation abilities. These deficits were more pronounced in BVP patients. Compared to healthy participants, both BVP and PPPD patients had a significant impairment of spatial orientation especially following passive whole‐body rotations without visual feedback, that is, a task that requires the correct integration of vestibular sensory information. This impairment in PPPD patients with normal vestibular function can be interpreted as a central suppression of vestibular input that is required in healthy individuals to continuously update the internal representation of body motion and position relative to the environment, and has implications for both PPPD diagnosis and therapy.

## Author Contributions

**Vivien Oertle**: conceptualization, investigation, writing – original draft, writing – review and editing, validation, formal analysis, data curation. **Sandra Becker‐Bense**: investigation, writing – original draft, writing – review and editing, validation, supervision, resources, project administration. **Thomas Brandt**: conceptualization, writing – original draft, writing – review and editing, validation, methodology, project administration, supervision. **Marianne Dieterich**: writing – original draft, writing – review and editing, conceptualization, validation, methodology, project administration, supervision, resources, funding acquisition. **Johannes Gerb**: conceptualization, investigation, writing – original draft, writing – review and editing, visualization, methodology, validation, formal analysis, project administration, data curation.

## Funding

The present study was supported by the Deutsche Stiftung Neurologie (DSN; project 80766113 to J.G. and M.D. and project 80721017 to M.D.) and the Deutsche Forschungsgemeinschaft (DFG, German Research Foundation) under Germany‘s Excellence Strategy (Munich Cluster for Systems Neurology: EXC 2145 SyNergy) to M.D. V.O. received additional funding through the LMU Munich as part of a doctoral scholarship.

## Conflicts of Interest

The authors declare no conflicts of interest.

## Peer Review

The peer review history for this article is available at https://publons.com/publon/10.1002/brb3.71025

## Data Availability

The raw data supporting the conclusions of this article will be made available by the authors, without undue reservation.
